# Molar pregnancy and childhood cancer: a population-based linkage study from Denmark

**DOI:** 10.1038/sj.bjc.6603931

**Published:** 2007-08-14

**Authors:** J Schüz, N Martinussen, T Lightfoot, E Roman, J F Winther

**Affiliations:** 1 Institute of Cancer Epidemiology, Danish Cancer Society, Strandboulevarden 49, DK-2100 Copenhagen, Denmark; 2 Epidemiology & Genetics Unit, Department of Health Sciences, The University of York, Area 3 Seebohm Rowntree Building, Heslington, York YO10 5DD, UK

**Keywords:** childhood cancer, childhood leukaemia, molar pregnancy, epigenetics, population-based cohort study

## Abstract

We observed a relative risk of 1.40 (95% confidence interval; 0.86–2.16) for cancers diagnosed under the age 20 in 6192 offspring of 3431 mothers with a molar pregnancy, indicating it is not a major determinant of childhood cancer.

The few established causes of childhood cancer, including various chromosomal anomalies, account for only a small proportion of the total (Little, 1999). There is, however, evidence that certain types of childhood cancer originate *in utero*, although the underlying biological mechanisms are unclear (Hjalgrim *et al*, 2002). Maternal characteristics and pregnancy-related events have therefore been the focus of much research (Roman *et al*, 2005, 2006). The population-based UK childhood cancer case–control study (UKCCS, 2000), which examined the obstetric records of mothers of 2962 childhood cancer cases aged 0–14 years and 4864 matched controls found a statistically significant 2.5-fold increased childhood cancer risk among mothers with a previous molar pregnancy (Roman *et al*, 2005, 2006), suggesting a common biological process. We therefore conducted a register-based cohort study in Denmark to examine cancer incidence in the offspring of women who had been diagnosed as having a molar pregnancy.

## MATERIALS AND METHODS

Women who had a molar pregnancy before 2005 were identified using the Danish National Hospital Register, which contains information for virtually all non-psychiatric hospital admissions in Denmark since 1977. Details of children born to these women were obtained from the National Central Population Register (CPR), which was established in 1968 with all citizens having unique personal identification numbers that permit linkage between registers. The follow-up for cancer incidence, determined by linking to the National Danish Cancer Registry which has been in operation since 1943 (Storm *et al*, 1997), was up to 31 December 2003, the most recent year with complete registration. Although there were no age restrictions for the linkage, we decided to include only cancers diagnosed before the age of 20 years in the analyses.

In our analysis, the number of observed cases was compared with those expected. Expected numbers were determined by multiplying the number of person-years of cohort members by the incidence rates of primary cancer in the general population of Denmark (excluding non-melanoma skin cancer), in sex-specific 5-year age groups and 5-year calendar periods of observation. Standardised incidence ratios (SIRs) were calculated by dividing the observed and expected numbers, and exact 95% confidence limits of the SIRs obtained on the assumption of a Poisson's distribution of the observed cancers (Breslow and Day, 1987). Additional analyses involved restricting the age of follow-up to 14 years and to children born after their mother's molar pregnancy, the latter being primarily for comparison with previously published data (Roman *et al*, 2006). Data were also stratified by sex.

## RESULTS

Approximately 1 million women gave birth between 1977 and 2005 from which 3431 women with at least one molar pregnancy were identified. In total, 7403 children were born to these women, 507 of which were excluded as they were born after 31 December 2003, the cut-off for linking to the cancer registry. The final cohort of 6896 children was followed up for cancer diagnosis until they were 20 years old or 31 December 2003, whichever came first, accruing 83 945 person-years under risk. Characteristics of the 20 children who were diagnosed with cancer within this period of time are shown in Table 1. Non-melanoma skin cancers were excluded (one case).

The overall SIR based on 20 observed cases *vs* 14.3 expected was 1.40 (Table 2). When the data were stratified by cancer type no substantial differences were observed; based on two cases, the SIR was highest for testicular cancer. Furthermore, there were no apparent differences between boys (SIR 1.49) or girls (SIR 1.28). When we restricted the age of diagnosis from 0 to 14 years of age, we observed an overall SIR of 1.10 (95% confidence interval (CI); 0.58–1.88). To compare our data directly with that previously published (Roman *et al*, 2006), we examined cancer risk in children aged 0–14 years old whose mothers had had a previous molar pregnancy. We observed SIRs of 0.87 (95% CI: 0.28–2.03; 5 observed/5.8 expected), 1.77 (95% CI: 0.48–4.53; 4/2.3), and 0.00 (95% CI: 0.00–5.48; 0/0.7), for all cancers, haematological cancers, and sarcomas, respectively.

## DISCUSSION

We have investigated the relationship between cancers diagnosed before age 20 years in the offspring of mothers who had at least one molar pregnancy. For all cancers combined, the estimated relative risk was 1.40 (95% CI; 0.86–2.16), which although not as strong as that observed elsewhere (Roman *et al*, 2006), suggests this is an area that might benefit from further investigation.

The major strengths of the present study are its population-based setting and the unbiased and objective identification and follow-up of cohort members. Indeed, the high-quality, complete registers that were used for the ascertainment of women with a molar pregnancy and identification and follow-up of their offspring for cancer, are an excellent framework for investigating such a hypothesis. However, although we included the whole childhood population of Denmark over a period of almost 30 years we still have a relatively small cohort. Despite this, there was sufficient statistical power in the study to detect a twofold risk increase.

Children born after 1977 but whose mothers had had their only molar pregnancy before this time were not covered in the cohort but were included in the calculation of the reference rates. However, since molar pregnancy is a rare event, the number of such children is a very small proportion of all cancer cases and therefore the misclassification results in a minor loss of statistical power rather than a bias in the risk estimation. In contrast to the UKCCS analyses (Roman *et al*, 2006) our age range was 0–19 years and children were included irrespective of whether their mother's molar pregnancy was before or after the pregnancy with the index child – the design of the UK study meant obstetric data were only available for reproductive events before the birth of the index child. We did, however, generate risk estimates according to the UK analytical model for direct comparison.

The association between molar pregnancy and childhood cancer raises the interesting possibility of a common aetiology. Indeed, Roman *et al* (2006) speculated about the potential involvement of epigenetic mechanisms in relation to both molar pregnancy and childhood cancer development. Both childhood cancer and hydatidiform mole (HMs) are rare conditions with around 1 in 600 children likely to develop cancer before they are 15 years old (Parkin *et al*, 1998) and HM likely to occur in 1 in 1500 pregnancies in the western world (Altieri *et al*, 2003). Hydatidiform moles generally arise from an abnormal fertilisation and have been associated with the de-regulation of imprinted genes (Altieri *et al*, 2003; Slim and Mehio, 2007). Thus, while epigenetics is not a new concept with respect to the pathogenesis of HM (Kajii and Ohama, 1977), for childhood cancer it is a relatively new and expanding area of research. Loss of imprinting has been implicated in a number of congenital syndromes, some of which, such as Beckwith–Wiedemann syndrome, predispose towards certain childhood cancers (DeBaun and Tucker, 1998). The observation that childhood cancer can in some cases originate *in utero*, combined with the knowledge that many imprinted genes have key functions in regulating embryonic development (Robertson, 2005) suggest that it may be similar or even the same epigenetic predisposition that gives rise to both these conditions.

Recent work has identified *NALP7,* part of the CATERPILLAR family of proteins that are involved in cellular inflammatory responses to infections processes, as the gene causing familial recurrent HMs (Tschopp *et al*, 2003; Murdoch *et al*, 2006). While its exact role in familial recurrent HMs are unknown, for example it has no established role in DNA methylation, there have been several possible biological mechanisms put forward (Slim and Mehio, 2007). The most interesting of these in relation to childhood cancer, in particular childhood leukaemia, is the involvement of NALP7 in the cellular immune response. An abnormal immune relationship between a mother and a fetus has been associated with irregular pregnancy outcomes such as HM and choriocarcinoma (reviewed by Slim and Mehio, 2007). Thus insight into the aetiology of some childhood cancers may be gained by investigating the immune function and response of mothers of children with such conditions.

Importantly from a public health perspective the lack of a strong association between molar pregnancy and cancer in children and teenagers confirms the fact that molar pregnancy is not a major determinant of cancer in the young. However, the issue of overlapping aetiology remains one to be investigated in future research.

## Figures and Tables

**Table 1:** Characteristics of 20 children who developed cancer before the age of 20 years in a Danish cohort of children born of mothers diagnosed with a molar pregnancy

**Diagnostic main group**	**Cancer diagnosis**	**Age at diagnosis (years)**	**Gender^a^**
Haematological	Acute lymphoblastic leukaemia	2	F
diseases	Acute lymphoblastic leukaemia	17	M
	Acute lymphoblastic leukaemia	18	M
	Acute myeloblastic leukaemia	1	M
	Hodgkin's disease	14	F
	Non-Hodgkin's lymphoma (clinical diagnosis)	14	M
	Non-Hodgkin's lymphoma	5	M
CNS tumours	Brain, undefined malignant tumour (clinical diagnosis)	7	M
	Brain, undefined malignant tumour (clinical diagnosis)	18	M
	Cerebellar astrocytoma	8	F
	Cerebellar medulloblastoma	2	M
	Cerebellar medulloblastoma	8	F
	Neurilemmoma (Schwannoma)	8	F
Sarcomas	Mesenchymal chondrosarcoma	16	F
	Rhabdomyosarcoma	1	M
Renal tumours	Wilm's tumour	6	M
Testicular tumours	Malignant teratoma	15	M
	Teratocarcinoma	17	M
Ovarian tumours	Androblastoma	5	F
Neuroendocrinal tumours	Neuroendocrine carcinoma	17	F

a M=male, F=female.

**Table 2:** Observed (obs) and expected (exp) numbers and SIRs for all and selected types of cancer in children of women with a molar pregnancy, followed-up in childhood and adolescence

**Primary cancer of child**	**Obs**	**Exp**	**SIR^a^**	**95% CI^a^**
All cancers	20	14.3	1.40	0.86–2.16
Haematological diseases	7	5.4	1.30	0.52–2.68
Leukaemias	4	3.9	1.02	0.27–2.61
CNS tumours	6	4.6	1.32	0.48–2.88
Sarcomas	2	1.7	1.17	0.14–4.22
Testicular cancer	2	0.5	4.41	0.53–15.9

a CI=confidence interval; SIR=standardised incidence ratios.
